# Frequent Gain and Loss of Functional Transcription Factor Binding Sites

**DOI:** 10.1371/journal.pcbi.0030099

**Published:** 2007-05-25

**Authors:** Scott W Doniger, Justin C Fay

**Affiliations:** 1 Computational Biology Program, Washington University School of Medicine, St. Louis, Missouri, United States of America; 2 Department of Genetics, Washington University School of Medicine, St. Louis, Missouri, United States of America; McGill University, Canada

## Abstract

Cis-regulatory sequences are not always conserved across species. Divergence within cis-regulatory sequences may result from the evolution of species-specific patterns of gene expression or the flexible nature of the cis-regulatory code. The identification of functional divergence in cis-regulatory sequences is therefore important for both understanding the role of gene regulation in evolution and annotating regulatory elements. We have developed an evolutionary model to detect the loss of constraint on individual transcription factor binding sites (TFBSs). We find that a significant fraction of functionally constrained binding sites have been lost in a lineage-specific manner among three closely related yeast species. Binding site loss has previously been explained by turnover, where the concurrent gain and loss of a binding site maintains gene regulation. We estimate that nearly half of all loss events cannot be explained by binding site turnover. Recreating the mutations that led to binding site loss confirms that these sequence changes affect gene expression in some cases. We also estimate that there is a high rate of binding site gain, as more than half of experimentally identified S. cerevisiae binding sites are not conserved across species. The frequent gain and loss of TFBSs implies that cis-regulatory sequences are labile and, in the absence of turnover, may contribute to species-specific patterns of gene expression.

## Author Summary

Research in the field of molecular evolution is focused on understanding the genetic basis of functional differences between species. Protein coding sequences have traditionally been the focus of these studies, as the genetic code enables a detailed study of the strength of selection acting on amino acid sequences. However, from the earliest cross-species sequence comparisons, it was clear that protein sequences among closely related species are too similar to explain the observed phenotypic diversity. This led to the hypothesis that the evolution of gene regulation has played a key role in generating diversity between species. The availability of numerous complete genome sequences has made it possible to begin testing this hypothesis. In this work, the authors use an evolutionary model to identify functional divergence within transcription factor binding sites, the core functional elements involved in gene regulation. Applying this model to the baker's yeast, *Saccharomyces cerevisiae,* and its three closest relatives, the authors find that a substantial fraction of the ancestral binding sites have been lost in a species-specific manner. In some cases the loss of the binding site creates gene expression differences that may be indicative of species-specific changes in gene regulation. This work provides a useful computational framework that will allow further study of the conservation of cis-regulatory sequences and their role in molecular evolution.

## Introduction

Changes in gene regulation have been found in a wide range of species and can have a meaningful impact on cell and organismal phenotypes [1,2]. A significant fraction of regulatory variation can be attributed to changes in cis-regulatory sequences [3–7]. Changes in cis-regulatory sequences have been tracked to transcription factor binding sites (TFBSs), insertion of transposable elements, and variation in tandem repeats, e.g., [8–12]. Although changes in trans-acting factors are also important, e.g., [13–15], the molecular basis of changes in gene regulation will often require a dissection of cis-regulatory sequence evolution.

A major challenge in studying the evolution of cis-regulatory sequences is translating divergence in cis-regulatory sequences to divergence in regulatory function. Although conservation of sequence is a strong indicator of conservation of function, cis-regulatory sequences that have maintained their regulatory function can diverge to the extent that they are unalignable [16–19]. On a finer scale, experimentally identified TFBSs are not always conserved across species [20–22], even in cases when expression is known to be conserved [23]. The complex relationship between divergence in sequence and divergence in function [24] implies that the evolution of cis-regulatory sequences cannot be understood without investigating the evolution of individual TFBSs.

The turnover of TFBSs provides a simple explanation for divergence in cis-regulatory sequences without a change in regulatory function. Under the binding site turnover model, the chance gain of a new binding site creates redundancy and can lead to loss of either the new or original site [21,23,25]. Evolutionary models suggest that many novel binding sites can be created by a stochastic mutational process and can potentially lead to the loss of existing sites [22,26–29].

Empirical evidence suggests that binding site turnover may be common. For example, the change in the position and orientation of binding sites within the *even-skipped* (*eve*) stripe 2 enhancer produces no change in embryonic patterns of expression between species, but chimeric enhancers composed from different species result in mis-regulation [23]. Furthermore, many experimentally identified binding sites have credible counterparts at close but not orthologous positions in other species [20–22]. Thus, the gain and loss of TFBSs is directly relevant to understanding conservation and divergence in cis-regulatory sequences in relation to their function.

Models of TFBSs must account for sequence variations that have no affect on function or fitness [30,31]. Sequence variability within binding sites can arise as a consequence of a lack of specificity at certain positions or as a consequence of multiple sequences having the same binding energy. The specificity or binding probability of transcription factors for different DNA sequences has been modeled using both statistical mechanics [30] and information theory [32]. However, the relationship between binding probability and function or fitness is often not known. The simplest assumption is that both function and fitness are linearly related to the probability of being bound, which is approximately a step function of binding energy [30,33,34].

The distinction between sequences that can function as a binding site and sequences that cannot is critical to identifying the gain, loss, or turnover of TFBSs. The use of a cutoff, even one based on binding probability, is problematic when trying to classify sequences close to the cutoff [35]. One solution is to compare the likelihood of evolution under a model of neutral evolution to a model of a conserved binding site. Given a collection of known binding sites, the position-specific equilibrium base frequencies can be used to measure the strength of selection [36] and calculate the likelihood of evolution under a binding site model [28,37]. By combining models of neutral evolution with those for conserved binding sites, the frequency of conserved binding sites relative to those that have been gained or lost can be estimated [35].

Because the gain or loss of binding sites in nonfunctional sequences is common [22,26–29], it is difficult to identify which gain or loss events are functional and affect fitness without additional data. One approach is to examine the gain and loss of experimentally identified binding sites. A previous study in Drosophila melanogaster found that 5% of Zeste binding sites, identified by chromatin immunoprecipitation, have been lost or gained across Drosophila species, based on deviations from a conserved binding site model [38]. However, nonfunctional sequences may often be bound without affecting gene expression [39], and changes in gene expression may not always affect fitness [40,41].

A phylogenetic approach provides a means of identifying loss of functional binding sites based on significant conservation in some species but loss of constraint in others. This approach was used to identify cis-regulatory sequences around single-minded 2 (*SIM2*) that were conserved in some but not all mammalian species [42]. Here, we have used a phylogenetic approach to examine the frequency at which functional TFBSs have been lost across the genomes of four *Saccharomyces* species. These species are sufficiently different that even the three closest species provide enough signal to identify individual binding sites by sequence conservation alone [43]. Using a probabilistic model of TFBS evolution [35] for 91 different transcription factors [44], we found a substantial fraction of binding sites are not conserved between species, and that these sequence changes, at least in some cases, affect gene expression.

## Results

### A Model to Identify Semiconserved Transcription Factor Binding Sites

**Figure 1:**
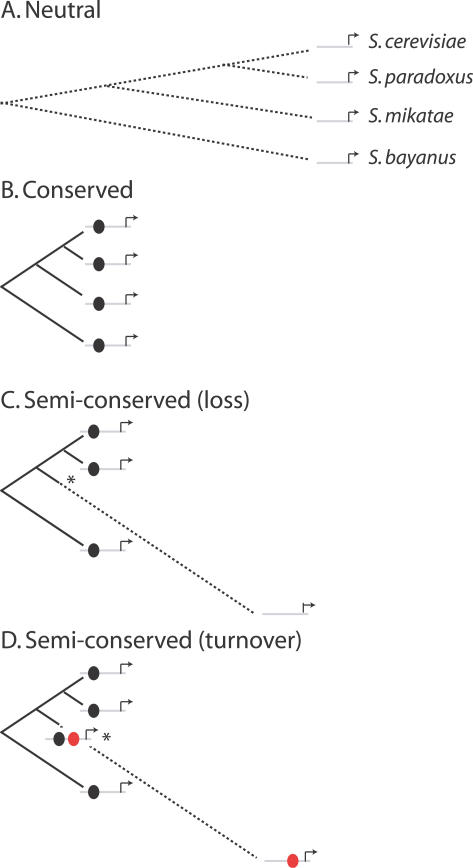
Evolutionary Models for Transcription Factor Binding Sites Three different evolutionary models are considered in this study: a neutral model of evolution, which assumes no functional constraint (A), a conserved TFBS model, which uses site-specific substitution matrices representing the varying constraints on each nucleotide position of a binding site (B), and a semiconserved model, which combines the neutral and TFBS models to identify sequences showing loss of constraint, indicated by the asterisk, (C). We also considered the case of loss in combination with gain, i.e., turnover (D), where the loss of an ancestral binding site (black oval) is accompanied by the gain of a compensatory binding site (red oval).

To identify semiconserved TFBSs, we used a probabilistic model of sequences evolving under a neutral and conserved binding site model. We define semiconserved sites as those that have been constrained along some lineages and unconstrained along others (Figure 1). Within this framework, semiconserved sites can be identified by their patterns of substitution rather than by their similarity to a binding site or a position weight matrix (PWM) representation of binding sites [45]. Additionally, semiconserved sites can be distinguished from conserved sites and neutrally evolving sequences by comparing the likelihood of a neutral model, a conserved binding site model, and a semiconserved model.

The likelihood of a set of aligned sequences under a neutral model or a conserved binding site model is a function of the substitution rate under each model. Under a binding site model, the substitution rate depends on position-specific functional constraints imposed by the sequence specificity of a transcription factor. At equilibrium, the expected frequency of a nucleotide base is a function of the strength of selection on the base relative to the other bases [36]. Thus, the equilibrium frequency of bases from a collection of binding sites can be used to estimate the intensity of selection and the expected rate of substitution at each position [46]. To compare the likelihood of evolution under a neutral and conserved binding site model, we used synonymous sites to estimate the neutral substitution rate and PWMs to estimate the equilibrium base frequencies within binding sites and derive position-specific substitution rates (see Methods).

The likelihood under a semiconserved model depends on which lineages have evolved under a neutral model and which have evolved under a binding site model. The semiconserved model can, in theory, detect both the loss and gain of binding sites. However, constraint on only a single lineage is typically indistinguishable from neutral evolution. Thus, we limited our analysis to loss of constraint on a single lineage and we did not consider loss events on the outgroup lineage. Since the lineage and time at which loss of constraint occurred is unknown, we calculated the likelihood under the semiconserved model by integrating over a large number of loss events evenly distributed over all lineages excluding the outgroup lineage, an approximation of the method used by Mustonen and Lassig [35].

### The Frequency of Semiconserved Binding Sites in Four *Saccharomyces* Genomes

To estimate the frequency of semiconserved relative to conserved binding sites, we used 91 TFBS models [44] and 1.7 megabases of noncoding sequences from 3,761 multiple sequence alignments of *S. cerevisiae, S. paradoxus, S. mikatae,* and S. bayanus [47]. Rather than test every position in the genome alignments, we calculated the likelihood under each model for the 2,000 positions with the highest-scoring sequence match to each binding site model in any two of the four species (see Methods).

We used expectation maximization to obtain an overall estimate of the frequency of sites evolving under each model. We found that 55% of the sites are best explained by the conserved binding site model, 31% are best explained by the semiconserved model, and 14% by the neutral model. The frequency of neutral sites is arbitrary since we did not test all positions within the alignments. Of the non-neutral sites, one-third are better explained by a model that allows for loss of constraint along one lineage. However, this estimate includes many sites that are reasonably explained by all three models. Sequences that don't provide a close fit to the conserved or semiconserved model may be evolving under a similar, yet unknown, model and may be incorrectly annotated as a semiconserved binding site.

**Figure 2:**
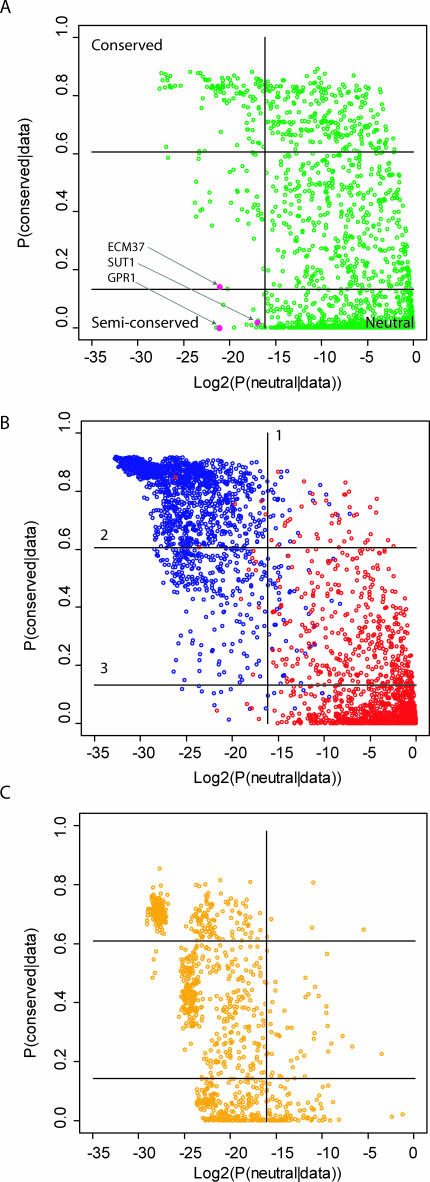
Identifying Conserved and Semiconserved Binding Sites (A) The distribution of posterior probabilities for 2,000 putative Rox1 binding sites present in yeast intergenic sequences. (B) The distribution of 2,000 Rox1 sites simulated under a neutral model (red) or a conserved binding site model (blue) as shown. (C) The distribution of 2,000 Rox1 sites simulated under a semiconserved model, where loss of constraint occurred at a random location on the phylogenetic tree, excluding the outgroup. The Log_2_ posterior probability of the neutral model is plotted on the *x*-axis, the posterior probability of the conserved model is plotted on the *y*-axis. Since the three probabilities sum to one, *p*(semiconserved | data) = 1 − *x* − *y*. Conserved and semiconserved sites were classified by three cutoffs (lines), defined in the text, and determined by the simulations. Sites passing cutoff one and two are annotated as conserved. Sites passing cutoff one and three are annotated as semiconserved. The three sites tested experimentally are shown in pink.

Figure 2A shows the posterior probabilities for 2,000 putative Rox1 sites present in the yeast genome alignments. Because the posterior probabilities sum to one, sites with a high likelihood under the semiconserved model but not the neutral or conserved model are shown in the bottom left corner, and sites with a high likelihood under the conserved model but not the neutral of the semiconserved model are shown in the upper left corner. The distribution of probabilities suggests that quite a few sites are equally well explained by each model.

To estimate our confidence in identifying individual sites that have evolved under a conserved or semiconserved model, and to eliminate sequences that may be evolving under a similar model, we generated null distributions for each model using computer simulations. Figure 2B shows the posterior probabilities for 2,000 sites simulated under a neutral model and 2,000 sites simulated under a model of a conserved Rox1 binding site. Three cutoffs were used to generate the high-confidence set of conserved and semiconserved sites (Figure 2B). The first cutoff delineates sites with a low probability under the neutral model (*p*(neutral) < 0.005). The second and third cutoffs delineate sites with a high probability under the conserved model and the semiconserved model, respectively. The second cutoff is set such that fewer than 1% of neutral sites show a higher likelihood under the conserved model. The third cutoff is set such that fewer than 1% of conserved sites show a higher likelihood under the semiconserved model.

Out of 2,000 putative Rox1 sites, 292 were inconsistent with a neutral model (cutoff 1, Figure 2A). Of these 292 sites, 242 sites were defined as conserved (cutoff 2) and 11 as semiconserved (cutoff 3). Out of 2,000 neutral simulations, two were defined as conserved and three were defined as semiconserved based on our cutoffs. Out of 2,000 conserved binding site simulations, 1% passed the semiconserved cutoff, suggesting that 292*1% ≈ 3 of the semiconserved sites are false positives. This data translates into a false discovery rate of 2/242 (1%) for conserved sites and 6/11 (54%) for semiconserved sites. The false discovery rates indicate that our cutoffs do not exactly produce a high-confidence set of semiconserved sites. However, simulations of semiconserved sites show the power to detect semiconserved Rox1 sites is only 16.4%, and increasing the stringency would reduce the power further (Figure 2C).

Using 91 TFBS models [44,48,49], we estimated the fraction of semiconserved sites for each. In total, we found 19,264 sites showed evidence of non-neutral evolution (*p* < 0.005 under the neutral model). Of these non-neutral sites, we classified 15,399 as conserved (*p* > 0.99 for the conserved model), and 982 as semiconserved model (*p* > 0.99 for the semiconserved model) (Table 1). In total, of the significantly conserved or semiconserved binding sites, 6.0% have been lost in a lineage specific manner. Semiconserved binding sites were identified for 85 out of 91 binding site models, and more than five loss events were found for 60 of the 91 models.

**Table 1:**
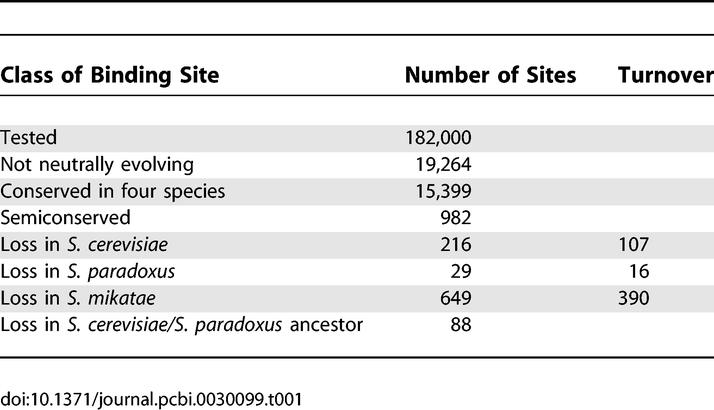
Number of Conserved and Semiconserved Binding Sites

To estimate the rate of false positive classification of conserved and semiconserved sites, we simulated 2,000 neutral and 2,000 conserved binding sites for each model. Classifying these simulated sequences, we found 224 neutral sequences passed our cutoffs for a conserved site and 242 neutral sites passed our semiconserved cutoffs. Thus, the rate of falsely classified conserved sites is just over 1% (224/15,399). By definition, 1% of the simulated conserved sites passed the semiconserved cutoff. Thus, the overall false discovery rate for semiconserved sites is 44% (19,264 * 0.01 + 224)/982.

### Characterization of Semiconserved Sites

The classification of sequences into conserved and semiconserved sites supposes that all sequences bound by the same protein evolve under the same functional constraints. However, for any given transcription factor, there may be certain sites in the genome that are selected for high binding energy and other sites that are selected for lower binding energy [33,35,50]. Selection for low-energy sites may produce the appearance of semiconserved sites if analyzed using a model based on high-energy sites.

**Figure 3:**
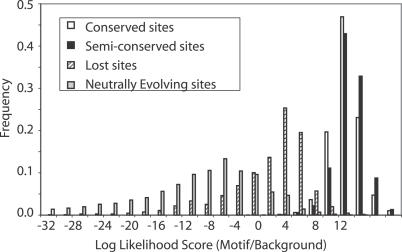
Distribution of Binding Site Scores from Neutral, Conserved, and Semiconserved Sites for 91 Binding Site Models We use the log-odds score of a sequence given a PWM relative to the genome-wide nucleotide frequencies as a proxy for binding energy. The semiconserved category (black bars) only includes sites from species where functional constraint has been maintained. The loss category (diagonally striped bars) shows sites from species where functional constraint has been lost. The neutral category (grey) shows sites generated by neutral simulations.

To investigate whether semiconserved sites may be low-energy binding sites, we examined the binding energies of conserved and semiconserved sites. We used the likelihood ratio score of a sequence under a binding site model compared with a model of background sequences as a proxy for binding energy [31]. The distribution of scores shows that semiconserved binding sites tend to have higher binding energy than the completely conserved sites on the lineages in which they have been conserved. In the lineage showing loss of constraint, the binding energies are much closer to background sequences (Figure 3). Additionally, the substitution rate within semiconserved sites is indistinguishable from that of conserved sites, excluding those lineages showing loss of constraint (Table 2). These comparisons suggest that semiconserved sites cannot be explained by a class of low-energy sites.

**Table 2:**
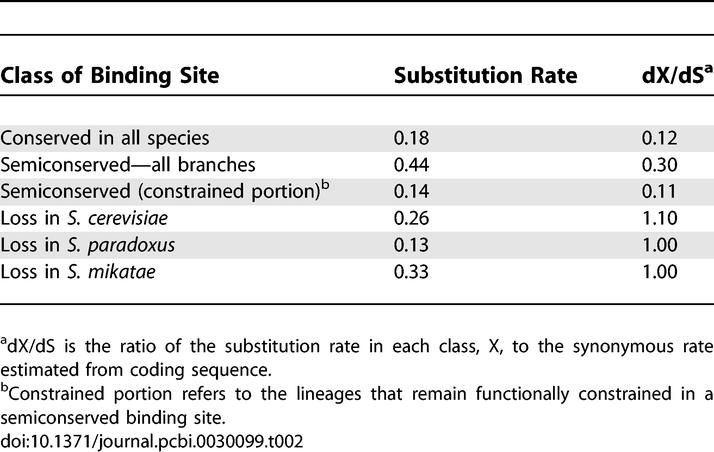
Substitution Rates in Conserved and Semiconserved Sites

### Evolution of Semiconserved Sites

Two models can explain the lineage-specific loss of TFBSs. First, some species may experience new environments where certain regulatory elements are not needed, or are selected against, resulting in a change in gene regulation. Second, the gain of one or more redundant binding sites within a promoter enables the loss of a previously constrained site (Figure 1D). Under the second model, the turnover of function from one binding site to another conserves the regulatory control but enables divergence within regulatory sequences.

The binding site turnover model predicts that binding site loss will be accompanied by the gain of a site elsewhere in the promoter. We tested this prediction by looking for the presence of a species-specific binding site for the same transcription factor in the promoter showing loss of constraint. We defined species-specific binding sites as a sequence that matches a PWM in one species, but whose orthologous sequences do not match the same weight matrix. To define a match to a PWM, we used a log-odds score cutoff from the tenth percentile score of conserved binding sites for each binding site model. Using this cutoff, 57% (513/894) of the species-specific loss events can be explained by turnover (Table 1). In comparison, species-specific sites are present within 50% of promoters with conserved sites and 47% of promoters with semiconserved sites, excluding lineages with loss. Using a more stringent cutoff score derived from information theory [45], 38% of the loss events can be explained by turnover.

Binding site turnover is not due to any one lineage or binding site model. The rate of turnover is similar across lineages, with 50% of sites showing turnover in *S. cerevisiae,* 55% in *S. paradoxus,* and 60% in S. mikatae. Although the rate of turnover varies across binding site models, most of this variation can be explained by the information content of the models and the size of the promoter sequences within which semiconserved sequences lie, consistent with previous work [28].

Natural selection may also result in lineage-specific loss of TFBSs. If the fitness effects of binding sites differ between species, bind sites may be lost without consequence or they may be selected against. However, it is also possible that semiconserved sites arise from compensatory changes that are more complicated than those described by a simple binding site turnover model. For example, binding site turnover may also occur between sites bound by different but functionally related transcription factors. Distinguishing between these two possibilities is not easy.

If some but not all species have undergone a substantial shift in selective pressures, binding site loss may show high rates on specific lineages. In contrast, if binding site loss is the result of turnover, loss should be a simple function of sequence divergence. The number of loss events on each lineage is heterogeneous (Table 1). Scaled by the synonymous substitution rate along each lineage, S. mikatae shows the greatest amount of loss, 66% of the loss events but only 40% of the total evolutionary distance, and S. paradoxus shows the least, 3% of the loss events but 16% of the evolutionary distance. However, simulations of semiconserved sites with loss events evenly distributed over the tree shows that the power of detecting binding site loss is the lowest on the shortest lineages, since these lineages have the fewest informative substitutions. One way to control for the confounding effects of power is to identify binding sites that show lineage-specific rates of loss that differ from the average lineage-specific rate across all binding site models.

Using the average rates of lineage-specific loss across all binding sites as a control (Table 1), we tested 29 binding site models with at least ten loss events for a heterogeneous distribution of binding site loss across lineages. We found significant heterogeneity in the loss of both Spt23 and Rlr1 binding sites (X^2^, 3 d.f., *p* = 3 × 10^−7^ for Spt23 and *p* = 4 × 10^−11^ for Rlr1). Spt23p stimulates Ty1 transposition and is a suppressor of Ty1-induced promoter mutations [51]. For Spt23 sites, the largest amount of loss was found on the lineage leading to S. paradoxus (14 loss events observed, 3.5 expected). Rlr1 is involved in transcription associated hyper-recombination between direct repeats [52]. For Rlr1, the largest amount of loss was found on the lineage leading to the ancestor of S. cerevisiae and S. paradoxus (38 loss events observed, 14.6 expected). The lineage-specific rate of loss of Spt23 and Rlr1 sites suggests that the loss of these sites may not have been a stochastic process.

### Substitutions Resulting in Binding Site Loss Cause Changes in Gene Expression

In the absence of binding site turnover, the semiconserved model predicts that the substitutions resulting in binding site loss should cause changes in gene expression. To experimentally determine whether semiconserved sites are functional and whether substitutions predicted to cause binding site loss are functional, we recreated the loss of three Rox1, two Ndt80, and six Msn2/4 semiconserved binding sites. These semiconserved sites were picked from 11, 14, and 27 semiconserved binding sites predicted using the Rox1, Ndt80, and Msn2/4 binding sites models, respectively.

**Figure 4:**
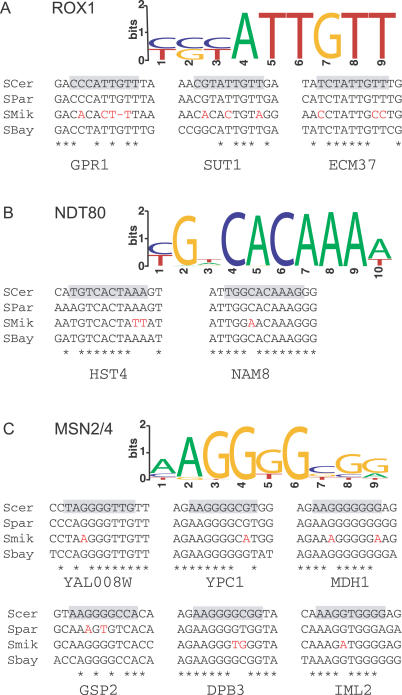
Semiconserved Binding Sites That Were Tested Using Gene Expression Assays The sequence logo representing the PWM and the alignment of each semiconserved binding site are shown for Rox1 (A), Ndt80 (B), and Msn2/4 (C). The binding site in S. cerevisiae is outlined in grey. The sequence changes shown in red were made in the S. cerevisiae promoter to test the predictions of the semiconserved binding site model.

For each semiconserved site, we used a beta-galactosidase reporter construct to compare the expression of the wild-type S. cerevisiae promoter with a mutated S. cerevisiae promoter containing the same substitutions predicted to cause change of function (Figure 4). Expression was measured in two strains of *S. cerevisiae,* one with and one without the transcription factor predicted to bind the site of interest.

Mutations in five of the 11 semiconserved binding sites affected levels of gene expression (Table 3). If these changes in expression are caused by the transcription factor predicted to bind the site, they should be absent in strains lacking the transcription factor. Using transcription factor deletion strains, we found that in only three of the five cases were these effects dependent on the presence of the transcription factor predicted to bind the site. Out of three semiconserved Rox1 binding sites, the site in the *SUT1* promoter showed a Rox1-dependent effect on gene expression. The three substitutions resulted in a 1.6-fold increase in gene expression, consistent with Rox1 being a transcriptional repressor in the presence of oxygen [53].

**Table 3:**
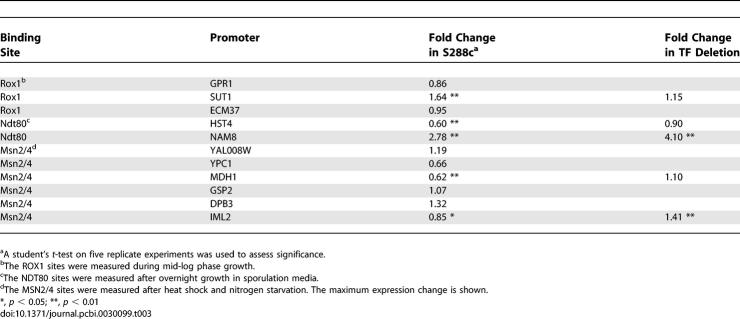
Substitutions Leading to Binding Site Loss Affect Gene Expression

Both of the semiconserved Ndt80 binding sites produced a significant effect on gene expression (Table 3). However, only in the *HST4* promoter is the effect dependent on Ndt80. The two substitutions in the *HST4* promoter led to a 1.7-fold decrease in gene expression during sporulation, consistent with Ndt80′s role as activating the middle sporulation genes [54]. In the *NAM8* promoter, a single substitution caused a 3-fold increase in expression during vegetative growth, independent of Ndt80.

Out of the six semiconserved Msn2/4 binding sites, the substitutions affected expression in two cases. Yet, of the two functional sites, only the one in the *MDH1* promoter affected expression in an Msn2–Msn4 double mutant (Table 3). Interestingly, this effect was only present during nitrogen starvation and not during heat shock.

### Limited Conservation of Experimentally Identified Transcription Factor Binding Sites

At equilibrium, the rate of binding site gain should be comparable to that of binding site loss. We previously showed that two Ndt80 binding sites, which show no conservation in other species, affected gene expression in S. cerevisiae [43]. Although the gain of a binding site that affects gene expression levels may be inconsequential to fitness, and thus susceptible to loss, the frequency at which functional binding sites are gained is relevant to understanding the evolution of gene regulation.

To estimate the rate of binding site gain across multiple transcription factors, we obtained a list of documented binding sites from the Yeastract database [55]. Because this database does not contain exact coordinates for each binding site, but rather transcription factor–promoter pairings, we limited our analysis to the 654 binding sites for 61 transcription factors where there was only a single high-scoring sequence match to the PWM in the promoter of interest. For each binding site, we tested its conservation across the four *Saccharomyces* species. We found that 303 (46.3%) of the Yeastract sites fit the conserved model, and seven (1.1%) fit the semiconserved model. Thus, a substantial fraction of experimentally identified binding sites appear to be species-specific or only weakly conserved across species, implying that binding site gain may be common.

## Discussion

Transcriptional regulatory sequences are expected to play an important role in molecular evolution. However, distinguishing functional from nonfunctional divergence within regulatory sequences continues to be a challenge. In this work, we have used a phylogenetic model to identify loss of constraint on individual TFBSs. Applying this model to four closely related *Saccharomyces* species, we found a substantial number of binding sites that show lineage-specific loss. In three out of 11 semiconserved sites tested, substitutions predicted to result in binding site loss affected gene expression levels in S. cerevisiae. Although a number of improvements can be made to models of TFBS evolution, there is considerable evidence for a continuous fine-scale rewiring of the transcriptional regulatory network at the level of individual promoters.

### The Rate of Binding Site Gain and Loss

The frequency of experimentally identified binding sites that are not conserved across species suggests a high rate of binding site gain. We found that more than half of the binding sites extracted from the Yeastract database [55] are not conserved. This is consistent with studies in other organisms. Between 30% and 50% of experimentally identified binding sites lie outside of conserved blocks in *Drosophila* [20], 40% of human and mouse TFBSs are species-specific [22], 5% of Zeste binding sites are not conserved among closely related *Drosophila* species [38], and 5% of CRP binding sites show presence and absence at orthologous positions in two bacterial genomes [35]. However, the biological relevance of these unconserved sites is not always known. Sites that are bound and affect gene expression may in some cases be lost without any fitness or downstream phenotypic consequences, except for a change in gene expression. In comparison, a binding site that has been conserved in some species but lost in others suggests that the site is relevant to fitness, at least in those species in which it was conserved.

The frequency of binding site loss may be quite high, but is difficult to estimate. Using expectation maximization, we estimated that one-third of all non-neutral sites are no longer constrained on some lineages. However, this estimate does not account for sequences that may have evolved under functional constraints other than the binding site model being tested. Using a number of statistical cutoffs to eliminate ambiguous sites, we found that 6% of the high-confidence binding sites fit the semiconserved model. This is similar to other estimates of the frequency of functional binding sites that are not entirely conserved across species [35,38]. Although some of these sites may be false positives, the true number of semiconserved sites could be higher, given that we estimated our power to detect semiconserved sites to be low, less than 20% for most models.

### The Effect of Binding Site Loss on Gene Expression

In the absence of binding site turnover, binding site loss results in species-specific changes in gene regulation. This model predicts that changes in gene expression should result from either making substitutions that result in loss in the species with a conserved site, or from making substitutions that recreate the binding site in the species showing loss. We tested the former of these two predictions using 11 different predictions of binding site loss. In three cases, we found that the substitutions predicted to result in loss of function altered the expression of the downstream gene. Although suggestive, these experiments do not address whether the substitutions that occurred on the lineage showing loss resulted in a species-specific change in gene regulation.

The eight of 11 semiconserved sites that showed no affect on gene expression are difficult to interpret. One explanation is that the semiconserved sites only affect gene expression under specific environmental conditions. Although possible, the gene expression assays were carried out under conditions where the semiconserved sites were likely to function. Another explanation is that our assays were not sensitive enough to detect small changes in gene expression. Finally, the predictions rest on the false positive rate of the model as well as on its assumptions. While it is difficult to distinguish between these possibilities, several pieces of evidence suggest that the assumptions of the model may not always be correct.

### Model Assumptions

Our predictions of binding site loss rest on a number of assumptions. The main assumptions are that the alignments are correct, the binding site models are correct, and that sequences that appear to be semiconserved binding sites are not functionally constrained for some other reason. We discuss each of these assumptions separately.

#### Alignments.

The incorrect alignment of one of the four species could make some conserved sites appear as though they were semiconserved. While incorrect alignments may occur, there are a number of reasons to believe that their impact in this dataset is negligible. Simulation studies show that the *Saccharomyces* species fall within the range where alignment algorithms perform well [56]. Additionally, realigning the ClustalW aligned sequences with Mlagan [57] leads to only 2% of the semiconserved sites being reannotated as conserved binding sites. Finally, we used local realignments surrounding the binding sites to eliminate mis-inference of loss caused by small insertion or deletion events (see Methods). It is also possible that turnover could be the result of misalignment. However, 54% of the turnover events occur in opposite orientation, making it unlikely to be the result of alignment error. Based on these data, we believe that the effects of alignment error in our analysis are likely to be small.

#### Position weight matrices.

The identification of binding site loss assumes that the binding site model is correct. Inaccuracies in the degeneracy of a binding site, or in the width of the binding site, will affect our results. One particular concern is that an overly specific binding site model will overestimate the rate of loss. PWMs are typically estimated from a subset of the true binding sites, and as a result of this sampling, a position might be defined as 90% A, when in actuality, A is only slightly favored over a T. As a result, an A to T substitution may result in a false prediction of binding site loss.

Three observations suggest that semiconserved sites cannot be completely explained by inaccuracies in the binding site model (see Protocol S1 for the methods). First, the distribution of lineage-specific substitutions that result in loss are evenly distributed across the nondegenerate positions within binding sites. Second, PWMs rebuilt to include nucleotide counts of both the conserved and semiconserved data still annotate half of the semiconserved sites as semiconserved, suggesting that these loss events cannot be explained by errors in the binding site model. Third, we repeated our analysis using a second set of binding site models [58] and estimated that 7.9% of the binding sites have been lost in a lineage-specific manner. These analyses further suggest that the exact PWMs used could be an important source of both false positives and false negatives, but that slight errors in the binding site models are unlikely to explain all of the loss events we have observed.

#### Functional overlap.

A third assumption is that the sequence conservation observed in a TFBS is the result of the constraints required to maintain the binding site rather than some other functional constraint. For example, conserved binding sites may appear to be semiconserved under similar binding site models. The observation that predictions of conserved binding sites often overlap [43] suggests that sequences may often be conserved for reasons other than the model used to identify them. In two of the eleven sites examined experimentally, we found changes in gene expression independent of the transcription factor predicted to bind them. This suggests that these noncoding sequences are functional cis-regulatory sequences, but are not bound by Ndt80 or Msn2/4. Overlapping predictions are unlikely to explain all of the semiconserved sites, as 75% of the semiconserved binding sites do not overlap any other known binding site model. Yet, we cannot rule out that other functional noncoding sequences, regulatory or otherwise, could be the basis of the functional constraint.

### The Molecular Evolution of cis-Regulatory Sequences

Although functional divergence in cis*-*regulatory sequences may be common, in relatively few cases have the nucleotide substitutions been identified [59]. TFBSs provide a useful starting point to dissecting sequence divergence that underlies regulatory divergence. The semiconserved model we have used in this analysis provides an efficient way to identify loss of constraint on a putative binding site sequence. Although several good candidate loss events were identified, there is a considerable false positive and false negative rate associated with the approach. Additional comparative information should help eliminate false positives, and methods that account for uncertainty in the binding site model should improve our ability to reliably detect functional divergence in cis-regulatory sequences.

## Materials and Methods

To distinguish neutral sequences from conserved and semiconserved binding sites, we used a model for the evolution of neutral sequence and functional TFBSs [35,37], calculated the likelihood of the data under three different evolutionary models, and used computer simulations to generate our statistical confidence in each model.

### Evolutionary models.

For each model, we assume that nucleotide sequences are evolving under a discrete-state, continuous-time Markov process, positions within an alignment evolve independently of one another, and the substitution rate is a product of the population size, *N*, mutation rate, *μ*, fixation probability, *f*, and time, *t*, measured in generations. We also assume that the mutational process is the same under each model and is governed by five parameters [60]: four parameters for the equilibrium nucleotide frequencies (*π_a_*, *π_c_*, *π_g_*, *π_t_*) and one parameter for the rate of transitions relative to transversions (*κ*).

The probability of fixation is different between the models. Under the neutral model, the probability of fixation is the same for all mutations. Under the binding site model, the relative probability of fixation between any two bases is:

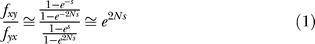
where *s* is the selective advantage of base *y* relative to base *x* [61]. The strength of selection can be estimated from the equilibrium base frequencies [36,62]. Given a collection of sites evolving under the same model, at equilibrium, the flux from base *x* to base *y* is equal to the flux from base *y* to *x*:

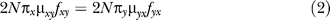
where *π_x_* is the equilibrium frequency of base *x*, *μ* is the mutation rate, and *f* is the fixation probability. Using the approximation of Equation 1, which assumes *Ns* > 1, and Equation 2, the equilibrium base frequencies are a simple function of the relative strength of selection and mutation:

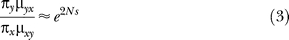



Substituting Equation 3 into Equation 1, the probability of fixation is:

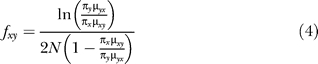
In the binding site model, the fixation probability is position-specific and derived from PWMs, as described below. Assuming the effective population size is constant, no estimate of *N* is needed since it is the same across all positions and all types of nucleotide changes.

### Calculating the likelihood.

We calculated the likelihood of the data under the neutral and conserved binding site model using transition probabilities derived from the expected rate of substitution under each model and using the pruning algorithm to integrate over all possible ancestral states [63]. To estimate the expected rate of substitution, we estimated *κ* from substitutions in synonymous sites in coding sequences (*κ* = 4), the *π* parameters from the genome-wide nucleotide frequencies (A = 0.3, G = 0.2, C = 0.2, T = 0.3) for the neutral model and from PWMs for each TFBS model. We estimated the mutation rate and time, together, for each branch of the phylogeny from synonymous sites using PAML [64]. Given these branch-specific substitution rates, we calculated the transition probability under each model by exponentiating the rate matrix, ***P*** = *e**^Q^***, where ***Q*** is a matrix of substitution rates of the form 2*Nμπft*.

For a pair of sequences, *x* and *y*, the likelihood of an aligned binding site, *S*, of width *W*, is given by:

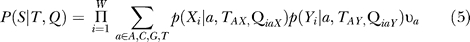



Here, *a* represents the nucleotide in the ancestral sequence *A*. *T_AX_* is the branch length from the ancestor to species *X*. *Q_iaX_* is the substitution rate from base *a* to base *X* in position *i*. *Q* can be either the neutral model of evolution (in which case it is position-independent), or the binding site model. *υ_a_* is the frequency of base *a* in ancestral sequence. For neutral sequence, this is the genome average frequency, *π_a_*. For the binding site model, this is the frequency of *a* in position *i* of the PWM. Equation 5 can be expanded to multiple sequences by recursively calculating the left and right branches of each node in the phylogenetic tree starting at the root [63].

To calculate the likelihood under the semiconserved model, we integrated over many loss events evenly distributed across the entire tree, excluding the outgroup. By re-rooting the tree at the time-point, *t*, where constraint was lost, we split the tree into two subtrees, with one subtree containing all sequences preceding *t*, and the other subtree with all sequences following *t*. The likelihood of the left and right subtrees was then calculated under the binding site model and the neutral model using the pruning algorithm and Equation 5. Thus, the likelihood under the semiconserved model is:

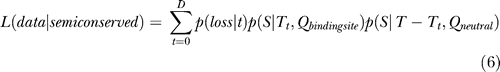
where *D* is the total evolutionary distance*, S* is the aligned binding site, *T_t_* is the portion of the tree evolving under the binding site specific model of evolution, *T − T_t_ i*s the neutrally evolving portion of the tree. Because very recent loss events are indistinguishable from the conserved binding site model, we do not test for loss events occurring within 0.1 substitutions per site of the extant species. We used the maximum-likelihood estimate of the location of the loss event to determine on which branch the loss of constraint occurred. Pseudocode can be found in Protocol S1.

### Maximum-likelihood estimate of the frequency of semiconservation.

To estimate the fraction of sites that are evolving under a semiconserved model of evolution, we used a maximum-likelihood approach. Using expectation maximization, we maximized the likelihood equation:

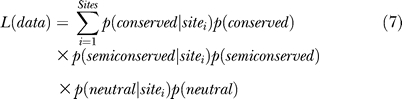

*p* (*conserved* | *site_i_*) and *p*(*neutral* | *site_i_*) were calculated using the pruning algorithm and Equation 5. *p*(*semiconserved* | *site_i_)* was calculated using Equation 6. *p*(c*onserved*), *p*(s*emiconserved*), and *p*(n*eutral)* are the free parameters that were maximized.

### Statistical cutoffs to distinguish between the models.

To distinguish between the three models, we compared the posterior probability of each model. While the maximum-likelihood estimates suggested that the probabilities of the three models are unequal, we used flat priors for simplicity. The choice of priors did change the overall annotations slightly, but the general conclusions are unchanged.

Computer simulations of neutral and conserved sequences were used to set statistical cutoffs for distinguishing each model and to estimate the power of detecting binding site loss. For each simulation, we evolved a sequence from the root of the tree to each node/tip using the transition probabilities specific to each model. For both simulations, we generated sequence at the root from the nucleotide frequencies defined by the PWM.

We used 10,000 neutral simulations to generate the neutral cutoff (#1 in Figure 2B), such that less than 0.5% of sites show a lower posteriori probability under the neutral model. The same data were used to generate the conserved cutoff (#2 in Figure 2B), such that less than 1% of neutral sites show a higher posteriori probability under the conserved model. We used 5,000 conserved binding site simulations for each transcription factor to generate the semiconserved cutoff (#3 in Figure 2B), such that less than 1% of sites show a lower probability under the conserved model.

To control the false discovery rate and computational time, we tested only the 2,000 highest-scoring binding sites for each transcription factor. To identify these sites, we ranked each putative binding site by the sum of the two highest-scoring sequences from the four species examined by their log-odds score, see below. The choice of 2,000 sites is arbitrary, but as most transcription factors are expected to regulate fewer than a few hundred genes, this should not exclude any functional binding sites from our analysis.

A summary table of the data for all 91 transcription factors can be found in Table S1. The genomic coordinates of all conserved and semiconserved binding sites are provided in Table S2.

### Applying the model to the *Saccharomyces* genomes.

*Alignments.* ClustalW intergenic sequence alignments of *S. cerevisiae, S. paradoxus, S. mikatae,* and S. bayanus [47] were filtered to remove any alignments containing greater than 50% insertions or deletions in any one sequence or those containing greater than 20% missing data (N and . characters). After applying these filters, global alignments of 3,761 intergenic sequences from four species were used in all subsequent analysis. 1,539 coding sequence alignments were used to estimate the synonymous and nonsynonymous substitution rates [64]. To account for insertion or deletion events within aligned binding sites, we generated local realignments by using the highest-scoring binding site in each species from the binding site and ±5 bp of it, excluding gaps.

*TFBS models.* We used the TFBS models defined by Harbison et al. [44], with the addition of a model for Ndt80 [48] and CSRE [49], as these well-studied motifs were not included. We filtered the dataset to remove dubious or redundant motifs, and used 91 out of 104 reported binding site models (see Table S1).

*Defining TFBS turnover.* We define binding site turnover as the presence of a species-specific binding site in the promoter of the species showing loss. To identify species-specific binding sites, we used the log-likelihood ratio score of the sequence given a PWM:

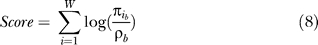
where *π_ib_* is the frequency of base *b* at position *i* of the binding site as defined by the PWM, *and ρ_b_* is the genomic frequency of base *b*, and *W* is the width of the binding site. To determine the cutoff score for a sequence match to the PWM, we used the distribution of scores in sites identified as significantly conserved. For each transcription factor, we enumerated the scores from all four species for each conserved binding site and used the tenth percentile of these scores to define a match to the PWM. To estimate the expected number of turnover events, we calculated the percentage of promoters containing a species-specific binding site for each transcription factor. We also used the default cutoff score of the Patser program [45] for comparison.

### Beta-galactosidase assays.

Beta-galactosidase activity driven by both a wild-type and mutant promoter sequence was measured to determine the effect of the binding-site loss on gene expression. For each putative loss event, the entire S. cerevisiae intergenic sequence was cloned by PCR with gap-repair or restriction digests into the YEp357r yeast–bacteria shuttle vector [65]. Mutations were made in the binding site to mimic the substitutions that occurred between species using stitching-PCR and were confirmed by sequencing. The constructs were transformed into the S. cerevisiae strain BY4743 or the appropriate homozygous deletion strain, obtained from the yeast deletion collection, for the transcription factor of interest [41]. The *msn2Δmsn4Δ* double-deletion strain was generated from a cross between the two single-deletion strains and confirmed by PCR.

To measure gene expression driven by either the S. cerevisiae binding site or the mutated binding site, yeast cultures were grown overnight in complete minimal medium minus uracil and diluted to a starting OD_600_ of 0.05. Each construct was measured in selective media during mid-log phase growth. The Ndt80 binding sites were also measured after 10 h in 1% potassium acetate. The Msn2/4 binding sites were also measured following a heat shock of 1 h at 37 °C, or 8 h in media with no nitrogen source.

The 11 sites tested were selected before the final statistical tests were applied. As a consequence, two of the 6 Msn2/4 binding sites, in *YAL008W* and *GSP2*, had a posteriori probability under the neutral model of 0.005 < *p* < 0.01.

### Yeastract data.

We downloaded the set of documented S. cerevisiae binding sites from the Yeastract database [55]. Because only transcription factor promoter pairings are reported (e.g., transcription factor X regulates gene Y), we limited the analysis to the 654 promoters with only a single high-quality match (greater than the 25th percentile of the log-odds scores of the conserved binding sites) to the transcription factor's binding site.

## Supporting Information

Protocol S1 Additional Methods and Pseudocode (49 KB DOC)

Table S1 Summary Table of the Results for Each of the 91 PWMs Examined (1.3 MB XLS)

Table S2 TFBS Annotations in General Feature Format (1.3 MB XLS)

## Abbreviations

PWM: position weight matrix
TFBS: transcription factor binding site
