# Correction: Frequent Gain and Loss of Functional Transcription Factor Binding Sites

**DOI:** 10.1371/annotation/363b6074-caec-4238-b88f-acbf45de498f

**Published:** 2009-02-05

**Authors:** Scott W Doniger, Justin C Fay

An incorrect version of Table S1 was published. The correct Table S1 file can be found here:
