# Supplementary material for: Frequent Gain and Loss of Functional Transcription Factor Binding Sites
Source: PLoS Comput Biol. 2007 May 25;3(5):e99. doi: 10.1371/journal.pcbi.0030099 (PMC1876492; doi:10.1371/journal.pcbi.0030099)
Supplement: Protocol S1 — (49 KB DOC) [file pcbi.0030099.sd001.doc]

**Protocol S1**

**Position weight matrix error**

The identification of binding site loss assumes that the binding site model is correct. While it is difficult to determine if a position weight matrix is correct, a number of observations suggest that inaccuracies in the binding site model cannot explain all of the binding site loss that we observed.

First, if a binding site model is overly specific, this over-specificity is likely limited to a small number of positions in the binding site. As a result, the substitutions that are the basis of the binding site loss prediction will be unequally distributed across the positions of the binding site. For each transcription factor we compared the distribution of the lineage-specific substitutions used to identify loss to the expected uniform distribution. We limit the analysis to only those positions with information content greater than 0.5 bits. In 4 out of 91 transcription factors (Phd1, Mot3, Spt2, and Sig1) there is a significant deviation from a uniform expectation (X^2^, p < 0.05). For example, Spt2 is an 11mer motif with 9 positions of high information content. Position 2 and position 7 account for 57% of the Spt2 loss events, suggesting that these two positions may be over specified. These four factors show an average of 11.5% loss and account for 124 of the loss events. Removing these four factors from the analysis lowers the estimated rate of loss to 5.5%.

Second, we have also tested for errors in the binding site model by rebuilding the PWM using nucleotide counts from both the conserved and semi-conserved predictions. In this way, we incorporate the loss events as allowable degeneracy into a new binding site model. By applying the model to the data from which it was built there is no sampling error and no over specification. If the original loss events are the result of inaccuracies in the original model, incorporating the loss events should correct the binding site model. For example, in position 3 of the Msn2/4 model we observed a high substitution rate in semi-conserved sites, with G to A substitutions appearing frequently under the original model. After rebuilding the Msn2/4 position weight matrix, a G to A substitution, while still expected to be rare, is now 30 times more likely along the branch leading to *S. mikatae* using the new matrix. When we re-annotated the original loss events with these models, 41% (11/27) of the Msn2/4 loss events remain annotated as highly significant loss events. An additional loss event is still significant at the less stringent 1% false positive rate. For Ndt80, 50% (7/14) of the loss events are still annotated as loss using the rebuilt PWM, and for Rox1, 64% (7/11) remain. Of the three experimentally confirmed semi-conserved sites, the Rox1 and Msn2 binding sites remain annotated as semi-conserved. The Ndt80 semi-conserved binding site is no longer a significant match to the semi-conserved model. After incorporating the loss events into the binding site model, we conclude that most of the loss events are due to substitutions in positions with stable, and therefore probably correct, nucleotide frequencies.

Third, to test the overall effects of the PWMs models used in this study, we repeated this analysis using a second set of binding site models [1]. Although these motifs are based on the same underlying chromatin immunoprecipitation dataset, they were defined using a different motif finding algorithm and provide a good test for the effect of the binding site models on our results. In total, using the 101 PWMs, and the same statistical thresholds, we identified 21,494 conserved binding sites, 1,758 semi-conserved sites, and estimate that 7.9% of the binding sites have been lost in a lineage-specific manner. The higher frequency of semi-conserved sites can be explained by the slightly higher information content and width of the MacIssac motifs which give us additional power to detect binding site loss. Additionally, we find that 51.7% of the loss events have been compensated for by turnover. We have also used these PWMs to reannotate the Yeastract binding site data, and find that 40.3% of the known binding sites are conserved and 1.7% are semi-conserved. While some of the annotations of individual binding sites do change between datasets, it is clear that the identification of semi-conserved binding sites is not specific to one set of PWMs.

These observations suggest that errors in the PWMs will affect our annotations, but also show that a substantial portion of the semi-conserved sites are unlikely to be explained by noise in the binding site models.

**Implementation of semi-conserved algorithm**

The equations outlined in the Methods section require a number of parameter choices. First, for the neutral model we used the HKY85 model [2]. We estimated the π parameters from the genomic nucleotide frequencies {A = 0.3, G = 0.2, C = 0.2, T = 0.3}, and used a transition/transversion ratio of 4, which was estimated from synonymous sites in coding sequences using PAML [3]. Additionally, the semi-conserved model requires a step size to integrate over loss events at different positions within the tree. We used a step size of 0.01 substitutions/site. The choice of step size did not affect the results, but does affect the computational time.

For a given PWM, we calculate the likelihood of the intergenic sequences under the neutral, conserved, or semi-conserved models using the following:

For each of the top 2000 putative binding sites S {

/*the top binding sites are predefined by ranking all sites by their two best log-odds scores from the 4 species. */

Neutral = 1;

Conserved = 1;

Semi = 1;

for i = 0 to PWM->width -1 {

n = 0;

c = 0;

for a in {A,C,G,T} {

n += f(a) * Felsenstein_prune (Root->left, Tree, S, i, Neutralmodel, a)

* Felsenstein_prune (Root->right, Tree,S, i, Neutralmodel, a);

//Neutralmodel is HKY85, Tree includes topology and branch lengths

c += PWM_i_(a) * Felsenstein_prune (Root->left, Tree, S, i, TFBSmodel, a)

* Felsenstein_prune (Root->right, Tree, S, i, TFBSmodel, a); //TFBSmodel is the scaled rate matrix (Equation 7).

}

Neutral += log(n);

Conserved += log(c);

Semi += log(Semiconserved (Tree, S, Neutralmodel, TFBSmodel, PWM))

}

report 3 likelihoods

}

}

sub Semiconserved (Tree T, Alignment S, Model N, Model TFBS, PWM pwm){

L = 1;

For i = 0 to pwm->Width {

totalp = 0;

For t = 0 to TotalDistance {

/*the branches are concatenated. If branch 1 is 0.1 subs/site long, t = 0.11 is the start of branch 2 */

Newtree = reroot (Tree, t);

p = 0;

For a in {A,C,G,T} {

p += PWM_i_(a) * Felsenstein_prune(NewRoot->left, S, i, TFBS, a)

* Felsenstein_prune (NewRoot->right, S, n, N, a);

}

totalP += Step_Size/TotalDistance * p;

}

L *= totalP;

}

return L;

//When when rerooting the tree only some of the branches within the original tree actually change their topology. Only those branched effected by rerooting need to be recalculated.

}

sub Felsenstein_prune (Node N, Alignment S, int n, Model M, Char a){

//use pruning algorithm with equation 2.

if (Node is a tip of the tree){

if (a == Observed sequence at node N in the phylogeny){

return 1;

else {

return 0;

}

else{

pLeft = 0;

pRight = 0;

For b in {A,C,G,T}{

pLeft += P(b | a, N->left_length, M) * Felesenstein_prune(N->left, S, n, M, b);

pRight += P(b | a, N->right_length, M) * Felesenstein_prune(N->right,S,n,M,b);

}

return pLeft*pRight;

}

}

1. MacIsaac KD, Wang T, Gordon DB, Gifford DK, Stormo GD, et al. (2006) An improved map of conserved regulatory sites for *Saccharomyces cerevisiae*. BMC Bioinformatics 7: 113.

2. Hasegawa M, Kishino H, Yano T (1985) Dating of the human-ape splitting by a molecular clock of mitochondrial DNA. J Mol Evol 22: 160-174.

3. Yang Z (1997) PAML: a program package for phylogenetic analysis by maximum likelihood. Comput Appl Biosci 13: 555-556.
